# The emerging role and clinicopathological significance of MFSD12 in cancer and lysosomal storage diseases

**DOI:** 10.3389/fphar.2024.1398320

**Published:** 2024-06-06

**Authors:** Liqiong Ding

**Affiliations:** Department of Pharmaceutics, School of Pharmacy, Hubei University of Science and Technology, Xianning, China

**Keywords:** MFSD12, lysosome, melanosome, melanoma, lysosomal storage diseases, PI3K

## Abstract

MFSD12 protein has recently risen as a key factor in malignancy and plays a potential role in a variety of complex oncogenic signaling cascades. Current studies suggest that MFSD12 has a positive complex role in the growth and progression of tumors such as melanoma, breast cancer, and lung cancer. At the same time, as a transporter of cysteine, MFSD12 is also involved in the development of lysosomal storage diseases. Therefore, MFSD12 may be an effective target to inhibit tumor development, block metastasis, and expand the therapeutic effect. This article reviews the molecular mechanisms of MFSD12 in a variety of cancers and lysosomal storage diseases.

## 1 Introduction

Semi-essential amino acid cysteine is required for the synthesis of numerous intracellular metabolites, including the powerful cellular antioxidant glutathione (GSH) (Stipanuk et al., 2006). Mostly found in the extracellular space as cystine, cysteine is actively taken up by the cell through the cystine-glutamate reverse transport system Xc^−^ (Conrad and Sato, 2012). Each cystine is converted to two cysteine molecules once it enters the cell. Studies have shown that most cysteine is imported into lysosomes via the major facilitator superfamily domain containing 12 (MFSD12) (Adelmann et al., 2020), and exported by transporter cystine (CTNS) to the cytosol to supply intracellular cysteine (Abu-Remaileh et al., 2017). Loss of function of CTNS can cause cystinosis, a lysosomal storage disease (LSD) (Gahl et al., 2002). In addition, lysosomal cysteine deficiency has been shown to lead to ferroptosis, a non-apoptotic cell death mediated by iron and peroxidation (Stockwell et al., 2017). Therefore, the maintenance of lysosomal cysteine homeostasis is very important.

MFSD12 is a newly discovered lysosomal cysteine transporter, which was first identified as a lysosomal protein involved in skin pigmentation (Crawford et al., 2017). It is reported that the decrease in MFSD12 expression was associated with a deepening of pigmentation and knockout of MFSD12 affects pigmentation in mice. In addition to its involvement in the regulation of skin pigmentation, in recent years, more and more studies have shown that MFSD12 affects the progression of various diseases such as LSD and cancer by participating in lysosomal cysteine transport. This article reviews the function of MFSD12 and its role in the occurrence and progression of diseases.

## 2 The structure and location of MFSD12

**FIGURE 1:**
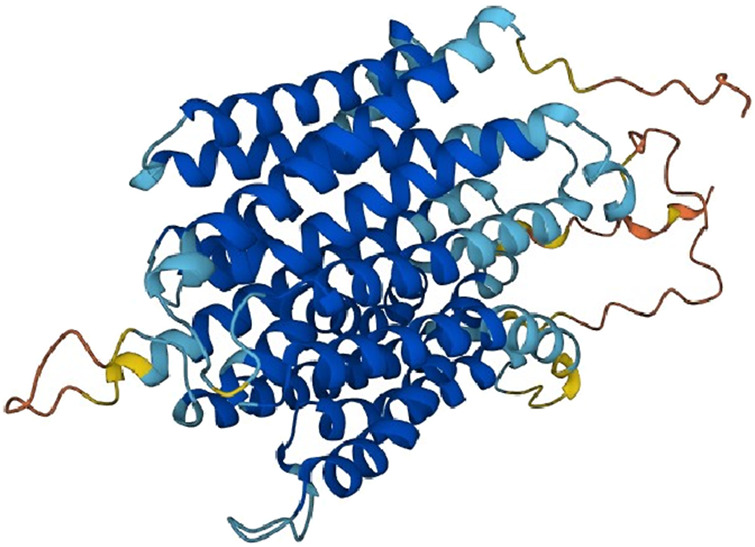
The structure of MFSD12.

MFSD12, located on chromosome 19, is a transmembrane protein predominantly localized to melanosomes and lysosomes, and is highly expressed in primary melanocytes (Figure 1). MFSD12 has homology with other genes that have MFS domains, and plays a role in transmembrane solute transport (Madej et al., 2013). MFSD12 mediates cysteine into melanosomes, thereby regulating transporters for skin pigmentation, and also mediates cysteine into lysosomes of non-pigmented cells (Yu et al., 2012; Adelmann et al., 2020).

## 3 The function of MFSD12

### 3.1 MFSD12 in cancer

#### 3.1.1 Melanoma

**FIGURE 2:**
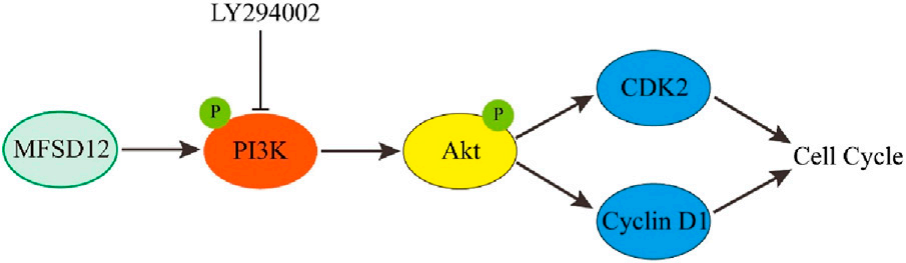
The role of MFSD12 in the progress of melanoma (Wei et al., 2019).

Melanoma is one of the deadliest skin malignancies (Rajabi et al., 2017). It was discovered that melanoma had a markedly and specifically raised expression of MFSD12, and that this protein stimulated cell cycle progression, therefore leading to increased cell proliferation (Wei et al., 2019). Mechanistically, PI3K signaling was triggered by MFSD12 overexpression, and the increase in cell proliferation that resulted from this activation was counteracted by PI3K inhibitors (Figure 2). Clinical research revealed a favorable correlation between reduced overall survival (OS) and disease-free survival (DFS) in melanoma patients and high expression of MFSD12. *In vivo* studies additionally verified that MFSD12 interference prevents lung metastasis of melanoma. Increased expression of MFSD12 leads to proliferation of melanoma cells, suggesting that MFSD12 is a useful prognostic biomarker and a potential therapeutic target for melanoma.

#### 3.1.2 Breast cancer

**FIGURE 3:**
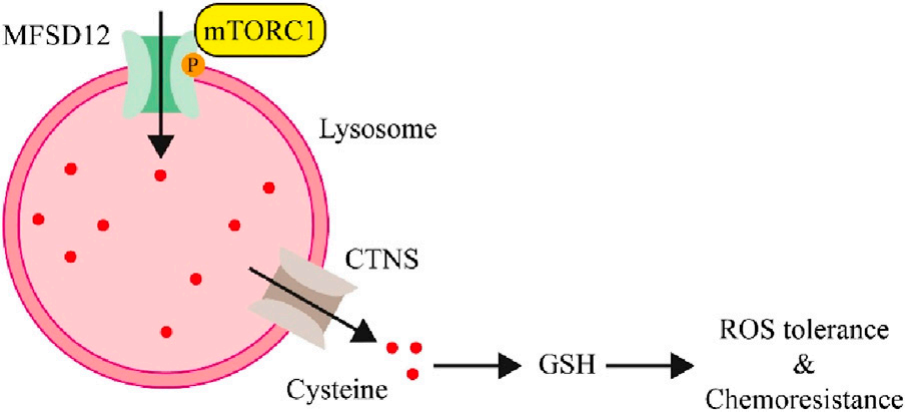
The involvement of MFSD12 in the treatment of breast cancer (He et al., 2023).

The production of GSH, which shields cancer cells from oxidative stress, requires cysteine as a necessary precursor (Daher et al., 2020; Bonifácio et al., 2021). He et al. discovered that MFSD12 was increased in breast cancer cells in order to enhance the storage of lysosomal cysteine, which is released by the CTNS in order to sustain GSH levels and mitigate oxidative stress (He et al., 2023). It was also shown that mTORC1 directly phosphorylates residue T254 to regulate MFSD12, and mTORC1 inhibits lysosomal acidification, which in turn activates CTNS (Figure 3). This switch adjusts redox homeostasis to improve cellular fitness and controls lysosomal cysteine levels in response to oxidative stress. The MFSD12-T254A mutant prevents tumor growth and MFSD12 function. Furthermore, in patients with breast cancer, MFSD12 overexpression was linked to unfavorable side effects and a worse prognosis from treatment. The findings point to MFSD12 as a possible therapeutic target of breast cancer and highlight the crucial role that lysosomal cysteine storage plays in adaptive redox homeostasis.

#### 3.1.3 Lung cancer

Within the tumor microenvironment, the most prevalent stromal cell group is known to be tumor-associated macrophages (TAMs). M1 TAMs are characterized as TAMs that suppress angiogenesis and stimulate anti-tumor immunity, while protumoral M2 TAMs are TAMs that encourage tumor growth, invasion, and metastasis (Zheng et al., 2017). Zheng et al. (2020) examined the association between TAM and overall survival as well as the density and distribution of TAM subtypes in the tumor core (TC) and invasive margin (IM) of human lung cancer. They found that lower M1 TC-TAM density and greater tumor cell proximity to M2 IM-TAM were linked to worse survival. In addition, RNA-seq results showed that M2 macrophages exhibited a considerable upregulation of MFSD12 in contrast to M1 macrophages. Furthermore, the decrease in MSFD12 expression level at TC in lung cancer patients was significantly associated with the increase in overall survival time. Shorter survival and lung metastases in melanoma patients have been found to be strongly linked with high MFSD12 expression (Wei et al., 2019). Therefore, MFSD12 is a promising marker of the macrophage subtype and a new predictive biomarker for lung cancer.

#### 3.1.4 Renal carcinoma

**FIGURE 4:**
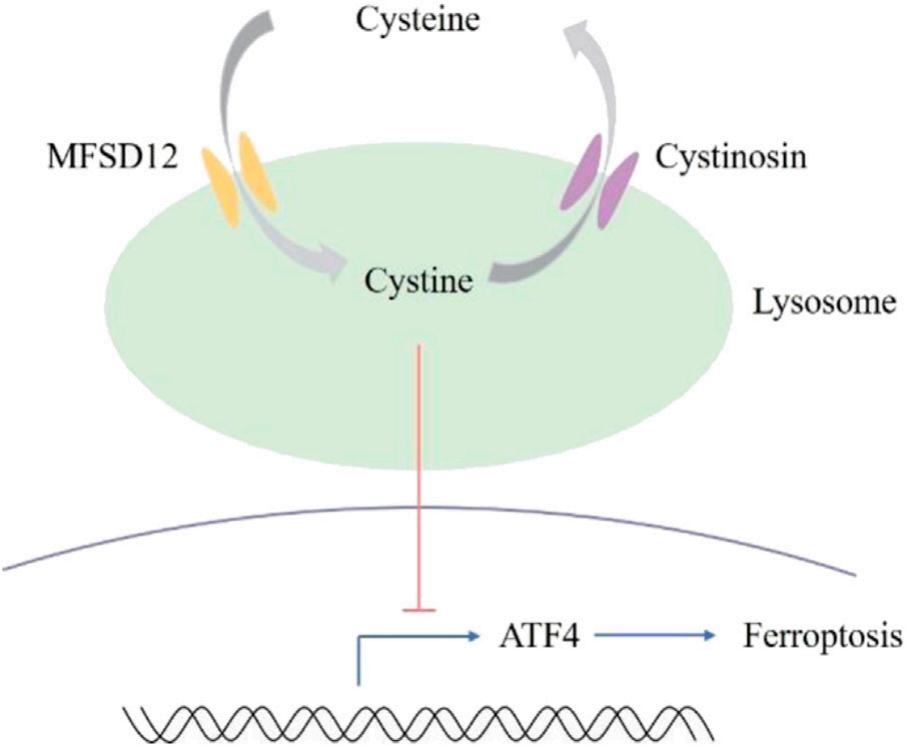
The involvement of MFSD12 and lysosomal cysteine in ferroptosis.

Ferroptosis, a type of non-apoptotic cell death mediated by iron and peroxidation, is caused by cysteine deprivation (Dixon et al., 2012; Stockwell et al., 2017). Important ferroptosis regulatory pathways in the cytoplasm and mitochondria, including dihydroorotate dehydrogenase (DHODH) (Mao et al., 2021), ferroptosis inhibitory protein 1 (FSP1) (Bersuker et al., 2019; Doll et al., 2019), and glutathione peroxidase 4 (GPX4) (Zhang et al., 2021), have been identified by recent investigations. Even though lysosomes are currently the signaling hubs of amino acid metabolism (Zhao et al., 2020), there is still a basic lack of understanding regarding the function of lysosomal cystine in ferroptosis. Numerous ferroptosis modulators have been investigated for their potential as therapeutic agents, and inducible ferroptosis has been employed in cancer treatment. However, the efficacy of ferroptosis in cancer is invariably offset by the adaptive ATF4 response. Thus, it would be ideal if therapeutic cysteine intervention could cause adaptive ATF4 to separate. Swanda et al. (2023) found that by means of the cysteine stress response, lysosomal cysteine regulates the sensitivity of ferroptosis in cancer. At the transcriptional level, ATF4 is induced by cysteine deprivation, and an adaptive ATF4 response is brought on by lysosomal cysteine insufficiency (Figure 4). Renal carcinoma cells, including UMRC6 cells and 786-O cells, become more susceptible to ferroptosis and ATF4 induction is attenuated when lysosomal cystine efflux is blocked. Knockdown of the cystine transporter cystinosin, which mobilizes lysosomal cystine to supply intracellular cysteine, almost completely inhibited the growth of UMRC6 tumors. However, the effect of MFSD12-mediated lysosomal cysteine entry on ATF4 expression and ferroptosis induction remains to be studied.

### 3.2 MFSD12 in lysosomal storage diseases

Previous studies have shown that inhibition of MFSD12 leads to dark pigmentation in mice and humans, but the specific mechanism is not well understood. Melanosomes are organelles associated with lysosomes that synthesize pigments (Dalba and Shawkey, 2019), and Adelmann et al. (2020) found that the production of cysteine dopa, a precursor in melanosomes that is synthesized by cysteine oxidation, and the maintenance of normal cysteine levels in melanosomes depend on MFSD12. Cysteine entrance into melanosomes and non-pigmented lysosomes requires MFSD12, according to tracing and biochemical analysis. Further studies found that in individuals with cystinosis, a lysosomal storage disease brought on by the inactivation of the cystine-exporting proteins in lysosomes, deletion of MFSD12 decreased the buildup of cystine in the lysosomes of fibroblasts (Town et al., 1998). Since MFSD12 is crucial to cysteine input by lysosomes and melanosomes, MFSD12 inhibitors may offer a novel class of drugs for the treatment of cystinosis.

Lipid-binding proteins assist lysosomal hydrolases in regulating the catabolism of sphingolipids (GSLs). The onset of lysosomal storage disease, which is typified by an abnormal accumulation of GSLs, is caused by disruption of catabolic pathway (Breiden and Sandhoff, 2019). The accumulation of GSLs might be caused by genetic defects of catabolic enzymes in lysosomal lumen or some protein-coding genes on lysosomal membrane involved in endocytic system fusion and transport (Platt et al., 2012; Hannun and Obeid, 2018). However, it is yet unknown which lysosomal membrane proteins were involved in the metabolism of GSL. Hong et al. (2023) showed that MFSD12 regulates GSL catabolism by linking the mTOR-TFEB pathway to lysosomal homeostasis in relation to cysteine transport. Lysosome-localized MFSD12, which transports cysteine in a way dependent on the H^+^ gradient, may influence GSL metabolism, and then control the mTOR-TFEB pathway to maintain lysosomal homeostasis.

## 4 Conclusion

Previous studies have shown that MFSD12 mediates cysteine transport in lysosomes and plays a leading role in the control of lysosomal storage diseases and signaling pathways in a variety of cancers. Expression of MFSD12 activates downstream signaling pathways, such as PI3K/AKT, while silencing MFSD12 expression may inhibit downstream target proteins, potentially preventing disease progression. Functional analysis has shown that MFSD12 can be considered an effective target for the treatment of cancer.

Although studies have shown that MFSD12 stimulates cell cycle progression in melanoma, leading to increased cell proliferation (Wei et al., 2019), however, Bhardwaj et al. found that knockdown of MFSD12 leads to increased levels of basal lysosomal lipid peroxidation (LLP) in melanoma cells, leading to lysosomal membrane penetration (LMP) and mediating cell death (Bhardwaj et al., 2023), suggesting that cysteine entry into lysosomes via MFSD12 is required to rescue LLP, LMP, and lysosomal cell death. Therefore, the mechanism by which this gene regulates carcinogenesis is still unclear, and the results of this study need to be further verified. At the same time, a better understanding of the typical functional domains of MFSD12 is expected to facilitate the development of small molecule targeted inhibitors. Future research may promote MFSD12 as a key tool for diagnosing cancer, observing cancer progression, and assessing treatment efficacy.
